# Back-translation for discovering distant protein homologies in the presence of frameshift mutations

**DOI:** 10.1186/1748-7188-5-6

**Published:** 2010-01-04

**Authors:** Marta Gîrdea, Laurent Noé, Gregory Kucherov

**Affiliations:** 1 Laboratoire d'Informatique Fondamentale de Lille (Centre National de la Recherche Scientifique, Université Lille 1), Lille, France; 2 Institut National de Recherche en Informatique et en Automatique, Centre de Recherche Lille - Nord Europe, France; 3 French-Russian J-V Poncelet Laboratory, Moscow, Russia

## Abstract

**Background:**

Frameshift mutations in protein-coding DNA sequences produce a drastic change in the resulting protein sequence, which prevents classic protein alignment methods from revealing the proteins' common origin. Moreover, when a large number of substitutions are additionally involved in the divergence, the homology detection becomes difficult even at the DNA level.

**Results:**

We developed a novel method to infer distant homology relations of two proteins, that accounts for frameshift and point mutations that may have affected the coding sequences. We design a dynamic programming alignment algorithm over memory-efficient graph representations of the complete set of putative DNA sequences of each protein, with the goal of determining the two putative DNA sequences which have the best scoring alignment under a powerful scoring system designed to reflect the most probable evolutionary process. Our implementation is freely available at http://bioinfo.lifl.fr/path/.

**Conclusions:**

Our approach allows to uncover evolutionary information that is not captured by traditional alignment methods, which is confirmed by biologically significant examples.

## Background

### Context and motivation

In protein-coding DNA sequences, frameshift mutations (insertions or deletions of one or more bases) can alter the translation reading frame, affecting all the amino acids encoded from that point forward. Thus, frameshifts produce a drastic change in the resulting protein sequence, preventing any similarity to be visible at the amino acid level. For that reason, classic protein alignment methods, that rely on amino acid comparisons, fail to reveal the proteins' common origins in the case of divergence by frameshift.

Consequently, it is natural to handle frameshift mutations at the DNA level, by DNA sequence comparisons. Several papers, including [1-4] reported functional frameshifts discovered using classic alignment tools from the BLAST [5,6] family. In all cases, the DNA sequences were relatively well conserved, which allowed the similarity to remain detectable at the DNA level.

However, the divergence may also involve additional base substitutions, that can reduce the similarity of the diverged DNA sequences. It has been shown [7-9] that, in coding DNA, there is a base compositional bias among codon positions, that no longer applies after a reading frame change. A frameshifted coding sequence can be affected by base substitutions leading to a composition that complies with this bias. If, in a long evolutionary time, a large number of codons in one or both sequences undergo such changes, they may be altered to such an extent that the common origin becomes difficult to observe by direct DNA comparison.

In this paper, we address the problem of finding distant protein homologies, in particular when the primary cause of the divergence is a frameshift. We aim at being able to detect the common origins of sequences even if they were affected by an important number of point mutations in addition to the frameshift. Also, when dealing with sequences that have little similarity, we wish to distinguish between sequences that are indeed (distantly) related, and sequences that resemble by chance. We achieve this by computing the best alignment of DNA sequences that encode the target proteins, with respect to a powerful scoring system that evaluates point mutations in their context, based on codon substitution patterns. Our approach implicitly explores all the pairs of DNA sequences that can be translated into these proteins, which allows a wider vision on the match possibilities at the DNA level.

We designed and implemented an efficient method for aligning putative coding DNA sequences, which builds expressive alignments between hypothetical nucleotide sequences obtained by back-translating the proteins, that can provide some information about the common ancestral sequence, if such a sequence exists. We perform the analysis on memory-efficient graph representations of the complete set of putative DNA sequences of each protein. The proposed method consists of a dynamic programming alignment algorithm that computes the two putative DNA sequences that have the best scoring alignment under an appropriate scoring system.

### Protein back-translation

Back-translation or reverse translation of a protein usually refers to obtaining one of the DNA sequences that encodes the given protein. Several methods for achieving this exist [10,11], aiming at finding the DNA sequence that is most likely to encode that protein. Several programs use multiple protein alignments to improve the back-translation [12,13]. This can be considered to be the opposite to the "translation way", where translation is used to improve coding DNA alignments or assess new coding DNA [14-17].

In this paper, we are not interested in just one of the coding sequences, but aim at exploring them exhaustively and aligning them with potential frameshifts. Thus, in the context of our work, the back-translation will refer to all the putative DNA sequences, as explained further in the **Methods **section.

### Similar approaches

The idea of using knowledge about coding DNA when aligning amino acid sequences has been explored in several papers.

A *non-statistical approach *for analyzing the homology and the "genetic semi-homology" in protein sequences was presented in [18,19]. Instead of using a statistically computed scoring matrix, amino acid similarities are scored according to the complexity of the substitution process at the DNA level, depending on the number and type (transition/transversion) of nucleotide changes that are necessary for replacing one amino acid by the other. This ensures a differentiated treatment of amino acid substitutions at different positions of the protein sequence, thus avoiding possible rough approximations resulting from scoring them equally, based on a classic scoring matrix. The main drawback of this approach is that it was not designed to cope with frameshift mutations.

Regarding *frameshift mutation discovery*, many studies [1-4] preferred the plain BLAST [5,6] alignment approach: BLASTN on DNA and mRNA, or BLASTX on mRNA and proteins, applicable only when the DNA sequences are sufficiently similar. BLASTX programs, although capable of insightful results thanks to the six frame translations, have the limitation of not being able to transparently manage frameshifts that occur inside the sequence, for example by reconstructing an alignment from pieces obtained on different reading frames.

For *handling frameshifts at the protein level*, [20] and [21] propose the use of 5 substitution matrices for aligning amino acids encoded on different reading frames, based on nucleotide pair matches between respective codons and amino acid substitution probabilities. One of the main differences between this scoring scheme and the one we present further in this paper is that our scores target nucleotide symbols explicitly, and are computed by taking into account the changes that occur at the DNA level directly. Also, our alignment method allows more flexibility with respect to frameshift gap placement within the alignment.

On the subject of *aligning coding DNA in presence of frameshift errors*, some related ideas were presented in [22,23]. The author proposed to search for protein homologies by aligning their *sequence graphs *(data structures similar to the ones we use in our method). The algorithm tries to align pairs of codons, possibly incomplete since gaps of size 1 or 2 can be inserted at arbitrary positions. The score for aligning two such codons is computed as the maximum substitution score of two amino acids that can be obtained by translating them. This results in a complex, time costly dynamic programming method that basically explores all the possible translations. Our algorithm addresses the same problem, by employing an approach that is more efficient, since it aligns nucleotides instead of codons, and works with simpler data structures thanks to the IUPAC ambiguity code, without any loss of information, as we will show further in the paper. Also, our alignment algorithm is more generic and is not restricted to a certain scoring function. Additionally, the scoring scheme we propose relies on codon evolution patterns, since we believe that, in frameshift mutation scenarios, the information provided by DNA sequence dynamics provides valuable information in addition to amino acid similarities.

## Methods

The problem of inferring homologies between distantly related proteins, whose divergence is the result of frameshifts and point mutations, is approached in this paper by determining the best pairwise alignment between two DNA sequences that encode the proteins.

Given two proteins *P*_*A *_and *P*_*B*_, the objective is to find a pair of DNA sequences, *D*_*A *_and *D*_*B*_, such that *translation*(*D*_*A*_) = *P*_*A *_and *translation*(*D*_*B*_) = *P*_*B*_, which produce the best pairwise alignment under a given scoring system.

The alignment algorithm incorporates a gap penalty that limits the number of frameshifts allowed in an alignment, to comply with the observed frequency of frameshifts in a coding sequence's evolution. The scoring system is based on possible mutational patterns of the sequences. This leads to reducing the false positive rate and focusing on alignments that are more likely to be biologically significant.

### Data structures: back-translation graphs

**Figure 1:**
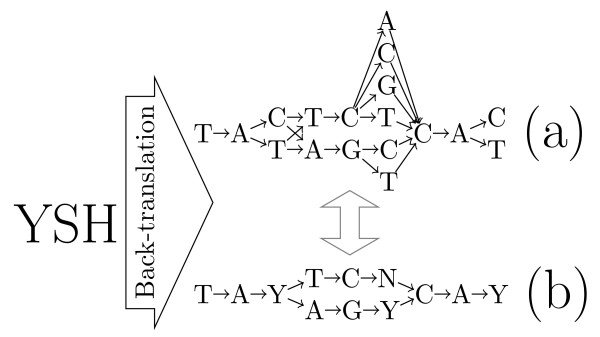
**Back-translation graph examples**. A fully represented (a) and condensed (b) back-translation graph for the amino acid sequence YSH.

An explicit enumeration and pairwise alignment of all the putative DNA sequences is not an option, since their number increases exponentially with the protein's length, as all amino acids are encoded by 2, 3, 4 or 6 codons, with the exception of *M *and *W*, which have a single corresponding codon. Therefore, we represent the protein's "back-translation" (set of possible source DNAs) as a directed acyclic graph, whose size depends linearly on the length of the protein, and where a path represents one putative sequence. As illustrated in Figure 1(a), the graph is organized as a sequence of length 3*n *where *n *is the length of the protein sequence. At each position *i *in the graph, there is a group of nodes, each node representing a possible nucleotide that can appear at position *i *in at least one of the putative coding sequences.

For identical nucleotides that appear at the same position of different codons for the same amino acid, and are preceded by different nucleotides within their respective codon, (as it is the case for bases *C *and *T *at the second position of the codons corresponding to amino acids *S *and *L *respectively), different nodes are introduced into the graph in order to avoid the creation of paths that do not correspond to actual putative DNA sequences for the given protein. Also, as the scoring system we propose in this paper requires to differentiate identical symbols by their context, identical nucleotides appearing at the third position of different codons for amino acids *L*, *S *and *R *will have different corresponding nodes in the back-translation graph. Basically, we can consider that each nucleotide symbol *α *from a putative coding DNA sequence, belonging to some codon *c*, is labeled with a word *l *which is its prefix in the codon *c*. Depending on the position of *α *in *c*, *l *will consist of 0, 1 or 2 letters. Here we denote such a labeled symbol by *α*^*l*^. Further in the paper we will drop the *l *for notation simplicity, and consider this differentiation implicit. Two symbols that appear at the same position of two putative DNA sequences encoding the same protein are identical (and are represented by the same node) if and only if they represent the same nucleotide and their labels are identical.

Two nodes at consecutive positions are linked by an arc if and only if they are either consecutive nucleotides of the same codon, or they are respectively the third and the first base of two consecutive codons. No other arcs exist in the graph.

**Figure 2:**
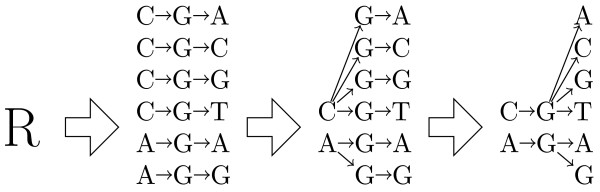
**Obtaining a simple back-translation graph for the amino acid *R***. The construction of a simple back-translation graph, for the amino acid *R*, encoded by 6 codons, is illustrated here. Note that identical nucleotides are associated to different nodes if they have different prefixes in the codons where they appear.

The construction of a simple back-translation graph for the amino acid *R *is illustrated in Figure 2.

More formally, a back-translation graph of an amino acid sequence *P *of length *n *is a directed acyclic graph *G*_*P *_= (*V*_*P*_, *E*_*P*_) where:(1)

where {} are the nucleotide symbols that appear at position *i *in at least one of the protein's putative coding sequences, and(2)

are arcs between nodes corresponding to symbols that are consecutive in one of the protein's putative coding sequences.

Note that, in the implementation, the number of nodes is reduced by using the IUPAC nucleotide codes [24]. For back-translating an amino acid, only 4 extra nucleotide symbols - *R*, *Y*, *H *and *N*, representing the sets {*A*, *G*}, {*C*, *T*}, {*A*, *C*, *T*} and {*A*, *C*, *G*, *T*} respectively - are necessary. In this condensed representation, the number of ramifications in the graph is substantially reduced, as illustrated by Figure 1. More precisely, the only amino acids with ramifications in their back-translation are amino acids *R*, *L *and *S*, each encoded by 6 codons with different prefixes, while the back-translations of all other amino acids are simple sequences of 3 symbols. As we will show below, there is no information loss regarding the actual pair of non-ambiguous symbols aligned.

**Figure 3:**
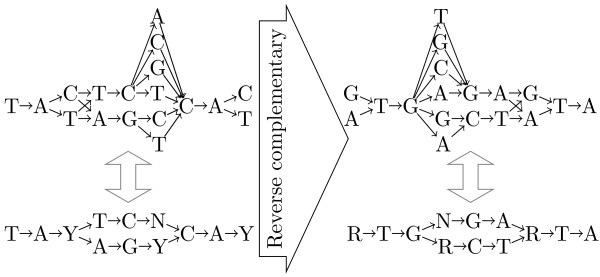
**Example of reverse complementary back-translation graphs for the amino acid sequence *Y SH***. The reverse complementary of a back-translation graph can be obtained in a classic manner, by reversing the arcs and complementing the nucleotide symbols that label the nodes.

The reverse complementary of a back-translation graph can be obtained in a classic manner, by reversing the arcs and complementing the nucleotide symbols that label the nodes, as illustrated in Figure 3.

### Alignment algorithm

When aligning two back-translated protein sequences, we are interested in finding the two putative DNA sequences (one for each protein) that are most similar. To achieve this, we use a dynamic programming method, similar to the Smith-Waterman algorithm [25], extended to back-translation graphs, and equipped with gap related restrictions.

**Figure 4:**
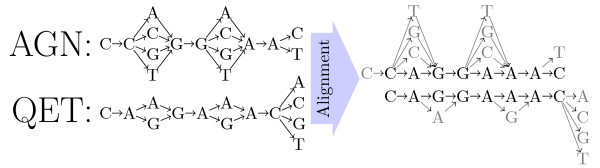
**Alignment example**. A path (corresponding to a putative DNA sequence) was chosen from each graph so that the match/mismatch ratio is maximized.

**Figure 5:**
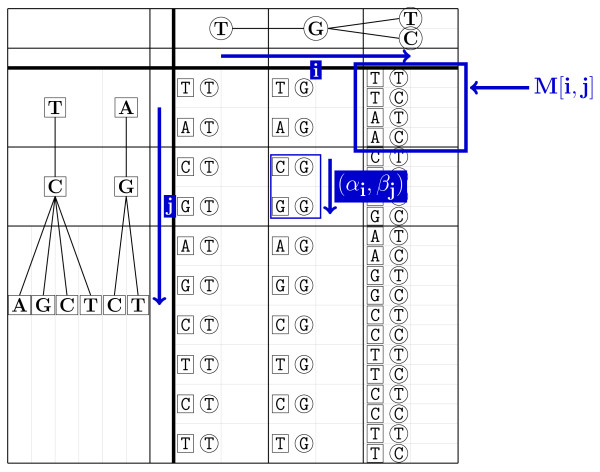
**Example of dynamic programming matrix *M***. *M *[*i*, *j*] is a "cell" of *M *corresponding to position *i *of the first graph and position *j *of the second graph. *M *[*i*, *j*] contains entries (*α*_*i*_, *β*_*j*_) corresponding to pairs of nodes occurring in the first graph at position *i*, and in the second graph at position *j*, respectively.

Given input graphs *G*_*A *_and *G*_*B *_obtained by back-translating proteins *P*_*A *_and *P*_*B*_, the algorithm finds the best scoring local alignment between two DNA sequences comprised in the back-translation graphs (illustrated in Figure 4). The alignment is built by filling each entry *M *[*i*, *j*, (*α*_*i*_, *β*_*j*_)] of a dynamic programming matrix *M*, where *i *and *j *are positions of *G*_*A *_and *G*_*B *_respectively, and (*α*_*i*_, *β*_*j*_) enumerates the possible pairs of nodes that can be found in *G*_*A *_at position *i*, and in *G*_*B *_at position *j*, respectively. An example of matrix *M *is given in Figure 5.

The dynamic programming algorithm begins with a classic local alignment initialization (0 at the top and left borders), followed by the recursion step described in relation (3). The partial alignment score of each matrix entry *M *[*i*, *j*, (*α*_*i*_, *β*_*j*_)] is computed as the maximum of 6 types of values:

(a) 0 (similarly to the classic Smith-Waterman algorithm, only non-negative scores are considered for local alignments).

(b) the substitution score of symbols (*α*_*i*_, *β*_*j*_), denoted *score*(*α*_*i*_, *β*_*j*_), added to the score of the best partial alignment ending in *M *[*i *- 1, *j *- 1], provided that the partially aligned paths contain *α*_*i *_at position *i *and *β*_*j *_on position *j *respectively; this condition is ensured by restricting the entries of *M *[*i *- 1, *j *- 1] to those labeled with symbols that precede *α*_*i *_and *β*_*j *_in the graphs, and is expressed in (3) by *α*_*i*-1 _∈ (*α*_*i*_), *β*_*j*-1 _∈ (*β*_*j*_).

(c) the cost *singleGapPenalty *of a frameshift (gap of size 1 or extension of a gap of size 1) in the first sequence, added to the score of the best partial alignment that ends in a cell *M *[*i*, *j *- 1, (*α*_*i*_, *β*_*j*-1_)], provided that *β*_*j*-1 _precedes *β*_*j *_in the second graph (*β*_*j*-1_∈  (*β*_*j*_)); this case is considered only if the number of allowed frameshifts on the current path is not exceeded, or a gap of size 1 is extended.

(d) the cost of a frameshift in the second sequence, added to a partial alignment score defined as above.

(e) the cost *tripleGapPenalty *of removing an entire codon from the first sequence, added to the score of the best partial alignment ending in a cell *M *[*i*, *j *- 3, (*α*_*i*_, *β*_*j*-3_)].

(f) the cost of removing an entire codon from the second sequence, added to the score of the best partial alignment ending in a cell *M *[*i *- 3, *j*, (*α*_*i*-3_, *β*_*j*_)]

We adopted a non-monotonic gap penalty function, where insertions and deletions of full codons are less penalized than reading frame disruptive gaps. Additionally, since frameshifts are considered to be very rare events, their number in an alignment is restricted. More precisely, as can be seen in equation (3), two particular kinds of gaps are considered: **i) frameshifts **- gaps of size 1 or 2, with high penalty, whose number in a local alignment is limited, and **ii) codon skips **- gaps of size 3 which correspond to the insertion or deletion of a whole codon.(3)

Although the algorithm is defined for back-translated protein alignment, it can also be used for aligning two DNA sequences or a DNA sequence to a protein. The graph corresponding to a DNA sequence has only one node at each position. Thus, the method can be used for aligning proteins to longer DNA sequences containing coding regions. However, when long sequences are aligned by dynamic programming methods, time and space complexity issues need to be addressed.

### Complexity and improvements

In this section we discuss the time and space complexity of our method and show how we can improve the latter using an approach inspired by [26].

#### Space complexity of the back-translation graphs

The space necessary for storing the back-translation graph of a protein sequence *P *of size *n *depends linearly on *n*. Basically, as mentioned in the section dedicated to data structures, the back-translation graph *G*_*P *_= (*V*_*P*_, *E*_*P*_) consists of 3·*n *groups of nodes {} (as each of the *n *amino-acids are encoded by sequences of 3 nucleotides). Every group *i *contains the nodes corresponding to the nucleotides that can appear at position *i *in at least one of the putative coding sequences (see (1)). The number of nodes in a group is limited by the number of codons that encode an amino acid, that we denote  (6 in the worst case scenario for non-ambiguous symbols) and thus does not depend on the protein's length.

Arcs exist only between nodes in consecutive groups (equation (2)), therefore each node can have a limited number of neighbors. Consequently, the overall memory consumption for storing the back-translation graph of a protein sequence *P *of size *n *is (*n*). The worst case scenario is a protein sequence composed only of the amino acids *L*, *S*, *R*, which are encoded by 6 codons each, and hence have the most complex back-translation. For each such amino acid, 10 nodes and 20 arcs are necessary, yielding a maximum memory size of 30*n *for the entire graph. For the ambiguous nucleotide symbol encoding though, 6 nodes and 6 arcs are necessary in the worst case for each amino acid, while most amino acids only require 3 nodes and 3 arcs for their back-translated representation.

#### Complexity of the alignment algorithm

Let *G*_*A *_and *G*_*B *_be graphs obtained by back-translating proteins *P*_*A *_and *P*_*B*_, of lengths *n*_*A *_and *n*_*B *_respectively. The dynamic programming matrix *M *computed by the alignment algorithm will have 3·*n*_*A *_+ 1 rows and 3·*n*_*B*_+ 1 columns. Each cell of the matrix *M *[*i*, *j*] has several entries corresponding to the possible pairs of nodes from each sequence. The number of entries is bounded by the square of the number of nodes that can appear on each position in the graph (^2^). Consequently, the total number of entries in the matrix is at most ^2^·(3·*n*_*A *_+ 1)·(3·*n*_*B *_+ 1), hence (*n*_*A*_*n*_*B*_).

Each entry holds the score of the partial alignment ending at the corresponding positions, as well as the number of frameshifts that occurred on the path so far (to ensure the established limit in the complete alignment) and a reference to the previous matrix entry of the alignment path, to facilitate the traceback. The storage space requirements for this supplementary information are bounded by a constant.

For computing each score in the matrix, the expressions that need to be evaluated are given by equation (3), by querying some of the entries from 5 other cells in the matrix. Since the number of entries in each cell is bounded by ^2^, this operation is considered to be performed in constant time. Consequently, the overall time complexity of the algorithm is (*n*_*A*_*n*_*B*_). To recover the best alignment and the two actual sequences that produce it, a classic traceback algorithm is used, with an execution time depending linearly on the alignment length, which cannot be larger than (3·*n*_*A *_+ 3·*n*_*B *_+ 1).

#### Improving the memory usage

To overcome the memory issues caused by aligning very large sequences with our dynamic programming method, which requires quadratic space, we used an approach inspired from the linear space algorithm for the LCS problem [26]. Our aim is to decrease the space consumption, not necessarily to linear space, with a less prominent increase of the computation time, i.e. the number of recursive matrix recomputations that are necessary for retrieving the actual alignment in this reduced space.

As a compromise, we choose to split the alignment according to some pre-established cut-points, in submatrices that are small enough to fit into memory, and that are recomputed only once for retrieving the corresponding alignment fragments. In our implementation, the cut-points delimit submatrices that are, by default, 128 columns wide. In this setup, we use a two-step approach: first, we compute the score of the best local alignment in linear space, using a sliding window, while also identifying the intersections of the corresponding path with the established cut-points; in the second step, we recompute separately the submatrices containing parts of the best alignment (restricted to the rows that intersect it), and then rebuild the alignment by pasting the obtained alignment fragments together.

For the first pass, we use a sliding window of 4 columns instead of the original 2, because each partial score depends on the scores that are 3 cells to the left or 3 cells above (see equation (3), items (e) and (f)). Each cell of the sliding window memorizes the matrix entry where the alignment path started (identified by the coordinates within the matrix and actual pair of aligned nodes), as well as the intersections of this path with the cut-points. This information is propagated from the previous cell contributing to the computation of the score, and completed in each cell from the cut-point columns by storing the line number and the node pair that help identify an actual entry in the matrix which belongs to the alignment path. The best scoring entry encountered so far is memorized and updated at each step of the alignment algorithm. When the first pass is completed, the best scoring cell will provide all the necessary information for reconstructing the alignment: the start of the alignment, the intersection with each cut-point, and its end, which is the cell itself. According to these coordinates, subgraphs of the two back-translation graphs are extracted and aligned globally (ensuring that the start and end node pair of each fragment are preserved). The obtained global alignments, combined, will give the best local alignment of the two large sequences.

### Translation-dependent scoring function

In this section, we present a new translation-dependent scoring system suitable for our alignment algorithm. Our scoring scheme incorporates information about possible mutational patterns for coding sequences, based on a codon substitution model, with the aim of filtering out alignments between sequences that are unlikely to have common origins.

Mutation rates have been shown to vary within genomes, under the influence of different factors, including neighbor bases [27]. Consequently, a model where all base mismatches are equally penalized is oversimplified, and ignores possibly precious information about the context of the substitution.

With the aim of retracing the sequence's evolution and revealing which base substitutions are more likely to occur within a given codon, our scoring system targets pairs of triplets (*α*, *p*, *a*), were *α *is a nucleotide, *p *is its position in the codon, and *α *is the amino acid encoded by that codon, thus differentiating various contexts of a substitution. There are 99 valid triplets out of the total of 240 hypothetical combinations. Pairwise alignment scores are computed for all possible pairs of valid triplets

(*t*_*i*_, *t*_*j*_) = ((*α*_*i*_, *p*_*i*_, *a*_*i*_), (*α*_*j*_, *p*_*j*_, *a*_*j*_)) as a classic log-odds ratio:(4)

where  is the frequency of the *t*_*i *_↔ *t*_*j *_substitution in related sequences, and  = *p*(*t*_*i*_)*p*(*t*_*j*_) is the background probability. This scoring function is used in the algorithm as shown by equation (3)(b), where we refer to it as *score*(*α*_*A*_, *α*_*B*_), without explicitly mentioning the context - amino acid and position in the corresponding codon - of the paired nucleotides. These details were omitted in equation (3) for generality (other scoring functions, that do not depend on the translation, can be used by the algorithm too) and for notation simplicity.

**Figure 6:**
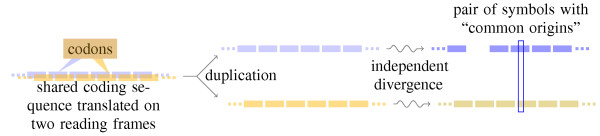
**Sequence divergence by frameshift mutation**. Two proteins are encoded on the same DNA sequence, on different reading frames; at some point, the sequence was duplicated and the two copies diverged independently; we assume that the two coding sequences undergo, in their independent evolution, synonymous and non-synonymous point mutations, or full codon insertions and removals.

In order to obtain the foreground probabilities , we consider the following scenario, depicted in Figure 6: two proteins are encoded on the same DNA sequence, on different reading frames; at some point, the sequence was duplicated and the two copies diverged independently; we assume that the two coding sequences undergo, in their independent evolution, synonymous and non-synonymous point mutations, or full codon insertions and removals.

The insignificant amount of available real data that fits our hypothesis does not allow classical, statistical computation of the foreground and background probabilities. Therefore, instead of doing statistics on real data directly, we will rely on codon frequency tables and codon substitution models, either mechanistic or empirically constructed.

#### Codon substitution models

##### Mechanistic codon substitution models

We can assume that codon substitutions in our scenarios are modeled by a Markov model presented in [28] that specifies the relative instantaneous substitution rate from codon *i *to codon *j *as:(5)

for all *i *≠ *j*. Here, the parameter *ω *represents the nonsynonymous-synonymous rate ratio, *κ *the transition-transversion rate ratio, and *π*_*j *_the equilibrium frequency of codon *j*. As in all Markov models of sequence evolution, absolute rates are found by normalizing the relative rates to a mean rate of 1 at equilibrium, that is, by enforcing  and completing the instantaneous rate matrix *Q *by defining  to give a form in which the transition probability matrix is calculated as *P *(*θ*) = *e*^*θQ *^[29]. Evolutionary times *θ *are measured in expected number of nucleotide substitutions per codon.

Note that there exist some more advanced codon substitution models, targeting sequences with overlapping reading frames [30]. However, such models do not fit our scenario, because they are designed for overlapping reading frames, where a mutation affects both translated sequences, while in our case the sequences become at one point independent and undergo mutations independently.

##### Empirical codon substitution model

The mechanistic codon substitution model presented above simulates substitutions with accurate parameters, but does not take into account the selective pressure and the resulting effects on the final codon conservation.

One of these effects, most commonly known and most observable in alignments of coding sequences, is the "third base mutation": in most cases, the encoded amino acid is not changed by a transition mutation of the codon third base; this is true in some cases of transversion mutations as well.

There are several other specific conservation families for groups of amino acids, as the *aliphatic *conservation (amino acids *L*, *I*, *V*) where corresponding amino acid codons share *T *at their second base. The last base is, within this group, almost a free choice, while the first has a large degree of freedom. It is thus expected to frequently observe the second *T *conserved on such codons when aligned with the *aliphatic *group. A similar phenomenon (however with a weaker frequency) appears for the subset (*A*, *S*, *T*) of the "small" amino acids, where the codons have in common the second base *C*.

In other chemically related amino acid groups, the succession of nucleotide substitutions at the codon level follows more complex paths, as it is the case for positively charged amino acids (*R*, *K*), aromatic amino acids (*F*, *Y*, *W*), etc.

Such different and complex conservation patterns are difficult to express and model with simple rules. As most of the matrices built for proteins, an empirical estimation gives a very good global approximation. In [31], the first empirical codon substitution matrix entirely built from alignments of coding sequences from vertebrate DNA is presented. A set of 17,502 alignments of orthologous sequences from five vertebrate genomes yielded 8.3 million aligned codons from which the number of substitutions between codons were counted. From this data, 64 × 64 probability matrices and similarity score matrices ("1-codon PAM") were computed. One can use these probability matrices as an alternative to the ones obtained using the mechanistic model.

#### Foreground probabilities

Once the codon substitution probabilities are obtained,  can be deduced in several steps. Basically, we first need to identify all pairs of codons with a common subsequence, that have a perfect semi-global alignment (for instance, codons *CAT *and *ATG *satisfy this condition, having the common subsequence *AT*; this example is further explained below). We then assume that the codons from each pair undergo independent evolution, according to the codon substitution model. For the resulting codons, we compute, based on all possible original codon pairs, *p*((*α*_*i*_, *p*_*i*_, *c*_*i*_), (*α*_*j*_, *p*_*j*_, *c*_*j*_)) - the probability that nucleotide *α*_*i*_, located at position *p*_*i *_of codon *c*_*i*_, and nucleotide *α*_*j*_, situated on position *p*_*j *_of codon *c*_*j *_have a common origin (equation (7)). From these, we can immediately compute, as shown by equation (8) below, *p*((*α*_*i*_, *p*_*i*_, *a*_*i*_), (*α*_*j*_, *p*_*j*_, *a*_*j*_)), corresponding to the foreground probabilities , where *t*_*i *_= (*α*_*i*_, *p*_*i*_, *a*_*i*_) and *t*_*j *_= (*α*_*j*_, *p*_*j*_, *a*_*j*_).

In the following,  stands for the probability of the event *codon c_*i *_mutates into codon c_*j *_in evolutionary time θ*, and is given by a codon substitution probability matrix (*θ*).

The notation **c**_i _[**interval**_i_] ≡ **c**_j _[**interval**_j_] states that codon *c*_*i *_restricted to the positions given by *interval*_*i *_is a sequence identical to *c*_*j *_restricted to *interval*_*j *_. This is equivalent to having a word *w *obtained by "merging" the two codons. For instance, if *c*_*i *_= *CAT *and *c*_*j *_= *ATG*, with their common substring being placed in *interval*_*i *_= [2..3] and *interval*_*j *_= [1..2] respectively, *w *is *CATG*.

We denote by **p**(**c**_i _[**interval**_i_] ≡ **c**_j _[**interval**_j_]) the probability to have *c*_*i *_and *c*_*j*_, in the relation described above, and we compute it as the probability of the word *w *obtained by "merging" the two codons. This function should be symmetric, it should depend on the codon distribution, and the probabilities of all the words *w *of a given length should sum to 1. However, since we consider the case where the same DNA sequence is translated on two different reading frames, one of the two translated sequences would have an atypical composition. Consequently, the probability of a word *w *is computed as if the sequence had the known codon composition when translated on the reading frame imposed by the first codon, or on the one imposed by the second. This hypothesis can be formalized as:(6)

where (*w*) and (*w*) are the probabilities of the word *w *in the reading frame imposed by the position of the first and second codon, respectively. This is computed as the products of the probabilities of the codons and codon pieces that compose the word *w *in the established reading frame. In the previous example, the probabilities of *w *= *CATG *in the first and second reading frame are:

The values of *p*((*α*_*i*_, *p*_*i*_, *c*_*i*_), (*α*_*j*_, *p*_*j*_, *c*_*j*_)) are computed as:(7)

from which obtaining the **foreground probabilities **is straightforward:(8)

#### Background probabilities

The **background probabilities **of (*t*_*i*_, *t*_*j*_), , can be simply expressed as the probability of the two symbols appearing independently in the sequences:(9)

#### Substitution matrix for ambiguous symbols

Earlier we have shown how to compute the translation dependent scores for non-ambiguous nucleotide symbols. However, as mentioned in the section concerning data structures, we work with ambiguous nucleotide symbols, because their usage improves time and memory consumption while providing the same final results. The scores for ambiguous nucleotide symbol pairs are easily obtained as follows:(10)

where  is an ambiguous nucleotide symbol representing the possible nucleotides that can appear on position *p*_*i *_of the codons that encode the amino acid *a*_*i*_, and *set*__denotes the set of non-ambiguous nucleotide symbols represented by . Basically, the score of pairing two ambiguous symbols is the maximum over all substitution scores for all pairs of nucleotides from the respective sets.

By using ambiguous symbols, less triplets are formed for each amino acid when compared with the non-ambiguous symbol case. 17 amino acids can be anti-translated as tri-mers with just one ambiguous symbol per position, while the others have two alternatives each of the three positions. Therefore, there are 69 different triplets with ambiguity codes to be paired (as opposed to 99), which means more than twice less storage space necessary for the score matrix.

For the reconstruction of the non-ambiguous putative DNA sequences at traceback, the actual pair of nucleotides that have the highest substitution score from the sets corresponding to two paired ambiguous symbols is required. These are easily obtained for each pair of ambiguous symbols as(11)

#### Parametrization

In this section we have presented a general framework that helps to compute a translation dependent scoring function for DNA sequence pairs, parametrized by a codon substitution model and an evolutionary time measured in expected number of mutations per codon. We consider that the sequences evolve independently, and the distance is relative to the original sequence.

#### Score evaluation

The score significance is estimated according to the Gumbel distribution, where the parameters *λ *and *K *are computed with the method described in [32,33]. In the future, we aim at improving our estimation by using a computation method more suited for gapped alignments, such as [34].

We use two different score evaluation parameter sets for the forward alignment (where the two back-translated graphs that are aligned have the same translation sense) and the reverse complementary alignment (where one of the graphs is aligned with the reverse complementary of the other), because these are two independent cases with different score distributions.

In order to obtain a more refined evaluation of the alignments, we introduce (*λ*, *K*) parameters for estimating the score significance of alignment fragments inside which the reading frame difference is preserved. Therefore, there are eight (*λ*, *K*) parameters that help to evaluate the alignments (four for the forward alignment sense and four for the reverse complementary alignment sense):

• (*λ*_*FW*_, *K*_*FW*_) for the forward sense and (*λ*_*RC*_, *K*_*RC*_) for the reverse complementary sense respectively, that are used for evaluating the score of the whole alignment.

• (*λ*_+*i*_, *K*_+*i*_) for the forward sense and (*λ*_-*i*_, *K*_-*i*_) for the reverse complementary sense respectively, with *i *∈ {0, 1, 2} that are used for evaluating the scores of each alignment fragment within which the reading frame difference is preserved. This second evaluation aims at providing a measure of the actual contribution of each such fragment to the score of the alignment.

The parameters (*λ*_±*i*_, *K*_±*i*_) are estimated on alignments restricted to the respective reading frame difference, where further frameshifts are not allowed, while (*λ*_*FW*_, *K*_*FW*_) and (*λ*_*RC*_, *K*_*RC*_) are computed in a more flexible setup, where a limited number of frameshifts is accepted.

#### Behavior in the non-frameshifted case

In this section we discuss the behavior of the proposed scoring system when aligning protein sequences without a frameshift. Given their construction method, we expect the scores to reflect the amino acid similarities, but also to be influenced by similarities at the DNA level.

To evaluate how our scores, used in non-frameshifted alignments, would relate to the classic scoring systems used by biological sequence comparison methods, we first compute, for each scoring matrix *T *corresponding to an evolutionary distance *θ*, the expected score for each amino acid pair, as:(12)

Where(13)

Then, considering each amino acid pair as an observation, we compute the correlation coefficient of these expected scores and the BLOSUM matrices as given by [35].

We also evaluate the correlation with the expected amino acid pair scores obtained when the sequences are aligned using a classic nucleotide match/mismatch system. The latter expected amino acid pair scores are also obtained as weighted sums of scores, in a manner similar to the one described by equations (12) and (13), where the score for aligning two symbols has one of the three established values for match, transition mutation or transversion mutation. For these classic scores, we used the values +5, -3, -4 in the examples reported below, although we have not noticed any drastic changes when different sets of values are used. The obtained correlation coefficients are reported in Table 1.

**Table 1:** Correlation coefficients of the translation dependent scores used on non-frameshifted amino acids, with BLOSUM scores and classic DNA scores

	DNA	BLOSUM 62
**TDS_*M *_(0.1)**	**0.86**	0.52
**TDS_*M *_(0.3)**	0.81	0.50
**TDS_*M *_(0.5)**	0.77	0.50
**TDS_*M *_(0.7)**	0.75	0.50
**TDS_*M *_(1.0)**	0.71	0.47

**TDS_*E*_**	0.59	**0.88**

The correlation coefficients between several types of scores that can be used to align amino acids without a frameshift: i) expected amino acid pair scores obtained from codon alignment with a classic match/mismatch scoring scheme (denoted *DNA*); ii) expected amino acid pair scores obtained from the translation-dependent scoring matrices based on the mechanistic codon substitution model (denoted *TDS*_*M*_); iii) expected amino acid pair scores obtained from the translation-dependent scoring matrices based on the empirical codon substitution model (denoted *TDS*_*E*_); iv) BLOSUM matrices for amino acid sequence alignment.

They suggest that the obtained translation dependent score matrices, either obtained from mechanistic or empirical codon substitution models, are a compromise between the "fully selective" BLOSUM matrices and the non-selective DNA scores.

On the one hand, the scores obtained using the mechanistic model do not make use of the selective pressure, and for this reason are more likely to be correlated with the classic DNA scores. On the other hand, the scores based on empirical codon substitution models reflect the constraints imposed by the similarity of the amino acids encoded by the codons. Hence, they show a strong correlation with the BLOSUM matrices when used without a frameshift.

## Results and Discussion

We have proposed a method for aligning protein sequences with frameshifts, by back-translating the proteins into graphs that implicitly contain all the putative DNA sequences, and aligning them with a dynamic programming algorithm that uses a scoring system designed for this particular purpose.

### Implementation and availability

A Java implementation of our method is available at http://bioinfo.lifl.fr/path/. The files containing translation dependent score matrices computed for several evolutionary distances can be downloaded at the same address.

### Experimental results

We will further discuss several significant frameshifted alignments obtained with our method. The experimental results presented here were obtained in the following experimental setup: a search for frameshifted forward alignments was launched on samples from the full NCBI protein databases for several species, using a 00.50 base per codon divergence scoring matrix; we selected only the alignments with an E-value < 10^-9^, presenting at least one significant frameshift.

#### Yersinia pestis: Frameshifted transposases

**Figure 7:**
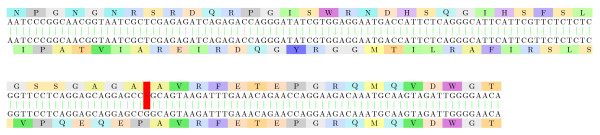
**Yersinia pestis: transposases**. The alignment of two transposase variants from *Yersinia pestis*: [GenBank:167423046] - subsequence 4-167 of the back-translation, and [GenBank:EDR63673.1] - subsequence 225-389 of the back-translation. The frameshift mutation at position 115/336 corrects the reading frame. The frameshifted alignment fragment has an E-value of 10^-7^.

Figure 7 displays the alignment of two transposase variants from *Yersinia pestis*. Both proteins are widely present on the NCBI nr database. The mechanism involved is (most probably) a programmed translational frameshifting since such mechanism has been quite frequently observed in several other transposases from related species, e.g. as in E. coli [36].

#### *Xylella fastidiosa*: Frameshifted *β*-glucosidases

**Figure 8:**
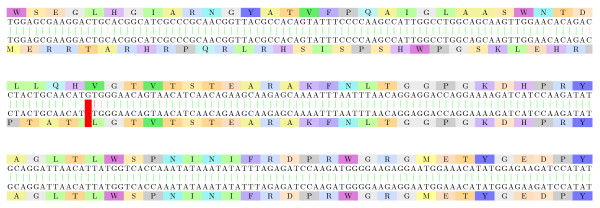
**Xylella fastidiosa: glucosidases**. Two *β*-glucosidase variants from *Xylella fastidiosa*: [GenBank:AAO29662.1] - subsequence 202-2645 of the back-translation, and [GenBank:EAO32640.1] - subsequence 2-2444 of the back-translation. We only show in this image a fragment of the full alignment (the first 239 base pairs). The second part is not particularly interesting in our context because the sequences are aligned on the same reading frame, with a very small number of mismatches. In the first part, the sequences are aligned with a reading frame difference that is corrected starting with positions 304/104. The frameshifted alignment fragment has an E-value of 10^-8^.

Two *β*-glucosidase variants from *Xylella fastidiosa *are aligned on Figure 8 with both variants widely present on the NCBI nr database. *Xylella fastidiosa *is a plant pathogen transmitted by *Cicadellidae *insects (*Homalodisca vitripennis*, *Homalodisca liturata*) and responsible for *phoney disease *on the peach tree, *leaf scorch *on the oleander, and *Pierce's disease *on grape. The *β*-glucosidase is usually required by several organisms to consume cellulose.

Interestingly, *β*-glucosidase frameshifts have already been studied in [37] on several bacteria including such *γ*-proteobacteria as *Erwinia herbicola *and *Escherichia coli *of *Enterobacteriales *but not directly observed in *Xanthomonadales*.

#### Venom neurotoxins

Diversification of venom toxins has been studied in [38] for advanced snakes: frameshifts were one of the most significant mechanisms involved in the "evolution of the arsenal" and its diversification toward specialized prey capture, sometime with a loss of neurotoxicity [39]. We thus studied neurotoxins from several higher snakes.

**Figure 9:**
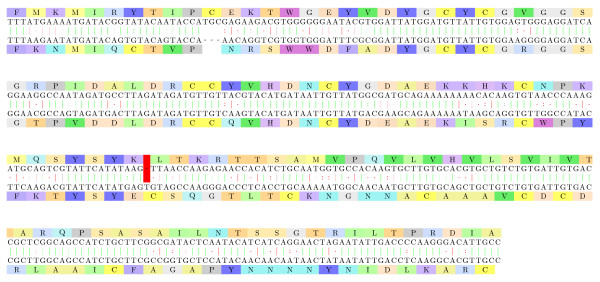
**Elapidae: neurotoxins (1)**. Two presynaptic neurotoxins from two higher snakes of the Elapidae family (*Bungarus candidus *and *Naja kaouthia*): [Swiss-Prot:Q8AY47.1] - subsequence 64-407 of the back-translation, and [PIR:PSNJ2K] - subsequence 13-354 of the back-translation. The sequences are aligned on the same reading frame up to position 186/135, and on a +1 reading frame from that point forward. The frameshifted fragment has an E-value of 10^-9^.

In Figure 9, we show the alignment of two presynaptic neurotoxins from two higher snakes of the Elapidae family (*Bungarus candidus *and *Naja kaouthia*). Most of the sites are conserved: the primary metal binding site and the putative hydrophobic channel remain before the frameshift, and only the fourth (and last) part of the catalytic network seems changed. We also noticed that, in the original second sequence, the Cysteine regions are more conserved at the DNA level than other amino acids, even after the frameshift, which is a strong hint of the non randomness of this part of the alignment.

**Figure 10:**
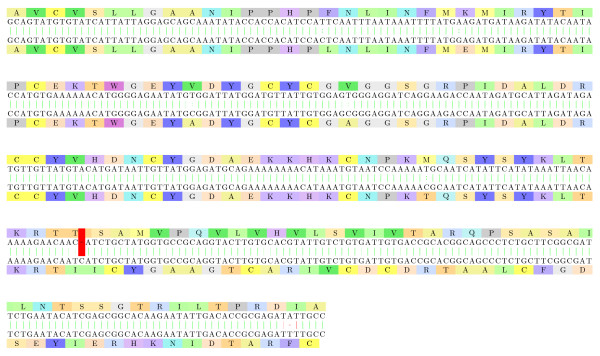
**Elapidae: neurotoxins (2)**. Two *Bungarus candidus *proteins, very similar at the DNA level ([Swiss-Prot:Q8AY47.1] and [Swiss-Prot:Q8AY48.1]). From the first 94 amino acid pairs, only 4 present mismatches (which are transitions at the coding DNA level). A frameshift mutation is visible at position 284 of the back-translated sequences. The fragments following it are almost perfectly aligned with a frameshift, with an E-value of 10^-9^.

Following the discovered frameshift of Figure 9, we took into consideration the sequences of *Bungarus candidus *species that were similar to the non-frameshifted presynaptic neurotoxin of *Naja kaouthia*. An interesting alignment is presented in Figure 10, showing a protein that aligns to it well but not perfectly (at least 4 non synonymous transitions before the frameshift and 1 transversion after): this lets open the potential "duplicated first then frameshifted" origin of the frameshifted protein. This assumption was strongly supported by the alignment of the two corresponding cDNA [GenBank:AY057881.1] and [GenBank:AY057880.1] of two homologous proteins.

**Figure 11:**
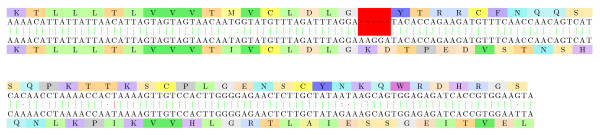
**Elapidae: neurotoxins (3)**. Two presynaptic neurotoxins from two higher snakes of the Elapidae family ([DDBJ:BAA75760.1] of *Laticauda colubrina *and [DDBJ:BAC78208.1] of *Laticauda laticaudata*): It shows that the unidentified peptide is in fact an alternative splicing (or frameshifted) variant of the neurotoxin. The frameshifted fragment has an E-value of 10^-10^.

Furthermore, Figure 11 displays an alignment of two presynaptic neurotoxins from two higher snakes of the Elapidae family (*Laticauda colubrina *and *Laticauda laticaudata*). It shows that the unidentified peptide is in fact an alternative splicing (or frameshifted) variant of the neurotoxin. Note that BLASTP identification of the frameshifted peptide on the NCBI nr database gives high E-values (minimal is of 0.67) and is thus expected to be missed by automatic prediction tools (since most of the features specific to neurotoxin are not present), whereas the non frameshifted peptide fragment, once identified, gives several E-values <10^-10 ^on NCBI nr.

#### Platelet-derived growth factor

**Figure 12:**
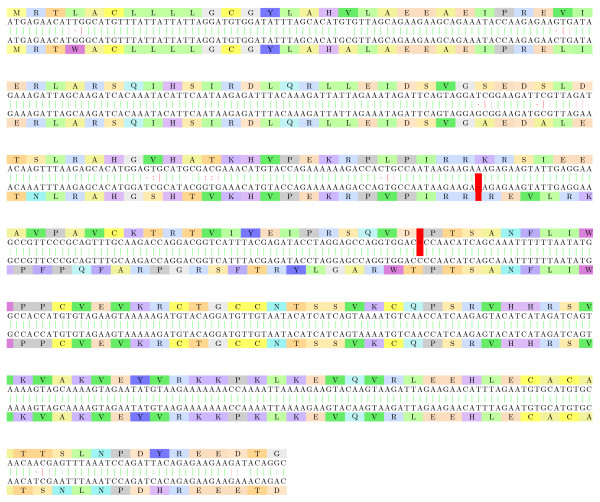
**Platelet-derived growth factor proteins**. The alignment of the back-translated platelet-derived growth factor proteins from *Homo sapiens *and *Ratus sp *([Swiss-Prot:P04085.1] and [DDBJ:BAA00987.1]). The two proteins share high similarity at the amino acid level on the subsequences 1-84 and 113-195. The amino acids 85-112 can be easily aligned with a frameshift, with an E-value of 10^-6^. Both the "inducing" and "correcting" frameshifts are located on two different exons.

**Figure 13:**
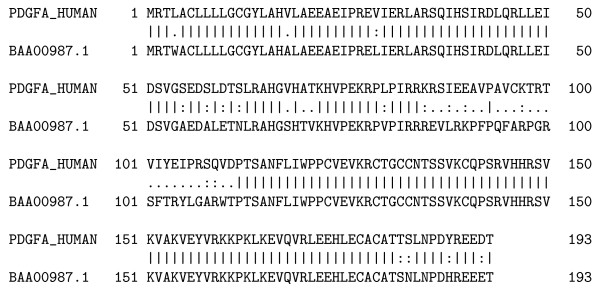
**Classic protein alignment of the platelet-derived growth factor proteins**. The classic protein alignment of the platelet-derived growth factor proteins from *Homo sapiens *and *Ratus sp *([Swiss-Prot:P04085.1] and [DDBJ:BAA00987.1]) shows very little amino acid similarity between the 85-112 subsequences, that we have successfully aligned on a +1 frameshift.

Platelet-derived growth factor is a potent mitogen for cells of mesenchymal origin. Binding of this growth factor to its affinity receptor elicits a variety of cellular responses. It is released by platelets upon wounding and plays an important role in stimulating adjacent cells to grow and thereby heals the wound. In Figure 12, we show the alignment of the back-translated *human *and *rat *platelet-derived growth factor proteins. The two proteins share high similarity at the amino acid level on the subsequences 1-84 and 113-195. The amino acids 85-112 can be easily aligned with a frameshift, as can be seen in Figure 12, while classic protein alignment reveals little similarity in these areas (Figure 13).

It is interesting to notice that this double frameshift (if confirmed) may have little influence on the protein (only the beginning of the receptor binding interface is modified). It is also interesting to notice that both the "inducing" and "correcting" frameshifts are located on two different exons.

## Conclusions

In this paper, we addressed the problem of finding distant protein homologies, in particular affected by frameshift events, from a codon evolution perspective. We search for protein common origins by implicitly aligning all their putative coding DNA sequences, stored in efficient data structures called back-translation graphs. Our approach relies on a dynamic programming alignment algorithm for these graphs, which involves a non-monotonic gap penalty that handles frameshifts and full codon indels differently. We designed a powerful translation-dependent scoring function for nucleotide pairs, based on codon substitution models, whose purpose is to reflect the expected dynamics of coding DNA sequences.

We illustrated our approach with several alignment examples of known and hypothetical frameshifted proteins, some of which are not detectable via classic alignment methods because of the low coding sequence similarity. Such examples support our method's applicability in the discovery of distant protein homologies and functional frameshifts, without any explicit information about the coding sequences. Future work includes further improvements of the scoring system, for example in order to focus on the detection of short double correcting frameshifts (two frameshifts separated by a small number of bases, where the second corrects the reading frame disrupted by the first). Such cases have been shown to occur frequently [37], but are often highly penalized by sequence comparison methods, that discard the correct alignment in favor of an ungapped one with a higher score.

Some natural extensions of our work include the support for multiple alignments of back-translation graphs. This feature can be useful for confirming frameshifts by similarity of the frameshifted subsequence with the corresponding fragments from several members of a family. Also, for boosting the efficiency, seeding techniques for back-translation graphs could be explored, possibly inspired by the BLASTP score-based seeds.

## Competing interests

The authors declare that they have no competing interests.

## Authors' contributions

GK initiated and guided the project, and proposed the first version of the method. MG refined the method, proposed the initial scoring system, did the implementation and the web interface. LN contributed to defining and improving the scoring system, and did most of the experimentation and the analysis of the results. MG drafted the manuscript, which was completed by LN with the "Experimental results" section, and then finalized and approved by GK.

## References

[B1] Raes J Peer Y Van de Functional divergence of proteins through frameshift mutations Trends in Genetics 2005 21 8 428 431 10.1016/j.tig.2005.05.013 15951050

[B2] Okamura K Feuk L Marquès-Bonet T Navarro A Scherer S Frequent appearance of novel protein-coding sequences by frameshift translation Genomics 2006 88 6 690 697 10.1016/j.ygeno.2006.06.009 16890400

[B3] Harrison P Yu Z Frame disruptions in human mRNA transcripts, and their relationship with splicing and protein structures BMC Genomics 2007 8 371 10.1186/1471-2164-8-371 17937804 PMC2194788

[B4] Hahn Y Lee B Identification of nine human-specific frameshift mutations by comparative analysis of the human and the chimpanzee genome sequences Bioinformatics 2005 21 Suppl 1 i186 i194 10.1093/bioinformatics/bti1000 15961456

[B5] Altschul S Gish W Miller W Myers E Lipman D Basic local alignment search tool JMB 1990 215 3 403 410 10.1016/S0022-2836(05)80360-2 2231712

[B6] Altschul S Madden T Schaffer A Zhang J Zhang Z Miller W Lipman D Gapped BLAST and PSI-BLAST: a new generation of protein database search programs Nucleic Acids Res 1997 25 17 3389 3402 10.1093/nar/25.17.3389 9254694 PMC146917

[B7] Grantham R Gautier C Gouy M Mercier R Pave A Codon catalog usage and the genome hypothesis Nucleic Acids Research 1980 8 49 62 10.1093/nar/8.1.197-c PMC327256 6986610

[B8] Shepherd JC Method to determine the reading frame of a protein from the purine/pyrimidine genome sequence and its possible evolutionary justification Proceedings National Academy Sciences USA 1981 78 1596 1600 10.1073/pnas.78.3.1596 PMC319178 6940175

[B9] Guigó R Bishop M DNA composition, codon usage and exon prediction Genetic databases 1999 53 80

[B10] Gonnet GH Back Translation (protein to DNA) in an optimal way Tech Rep 505 2005 Informatik, ETH, Zurich http://www.biorecipes.com/BackTranslate/code.html

[B11] Stothard P The sequence manipulation suite: JavaScript programs for analyzing and formatting protein and DNA sequences Biotechniques 2000 28 6 1102 1104 10868275 10.2144/00286ir01

[B12] Moreira A Maass A TIP: protein backtranslation aided by genetic algorithms Bioinformatics 2004 20 13 2148 2149 10.1093/bioinformatics/bth204 15059829

[B13] Giugno R Pulvirenti A Ragusa M Facciola L Patelmo L Di Pietro V Di Pietro C Purrello M Ferro A Locally sensitive backtranslation based on multiple sequence alignment Proceedings of the 2004 IEEE Symposium on Computational Intelligence in Bioinformatics and Computational Biology, (CIBCB) 2004 231 237

[B14] Suyama M Torrents D Bork P PAL2NAL: robust conversion of protein sequence alignments into the corresponding codon alignments Nucleic Acids Research 2006 34 Web Server W609 W612 10.1093/nar/gkl315 16845082 PMC1538804

[B15] Bininda-Emonds O transAlign: using amino acids to facilitate the multiple alignment of protein-coding DNA sequences BMC Bioinformatics 2005 6 156 10.1186/1471-2105-6-156 15969769 PMC1175081

[B16] Wernersson R Pedersen A RevTrans: Multiple alignment of coding DNA from aligned amino acid sequences Nucleic Acids Research 2003 31 13 3537 3539 10.1093/nar/gkg609 12824361 PMC169015

[B17] Fontaine A Touzet H Computational identification of protein-coding sequences by comparative analysis Proceedings of the 1st IEEE international conference on Bioinformatics and Biomedecine (BIBM), Silicon Valley, California 2007 95 102 10.1504/ijdmb.2009.024849 19517987

[B18] Leluk J A new algorithm for analysis of the homology in protein primary structure Computers and Chemistry 1998 22 123 131 10.1016/S0097-8485(97)00035-1 9570113

[B19] Leluk J A non-statistical approach to protein mutational variability BioSystems 2000 56 2-3 83 93 10.1016/S0303-2647(00)00074-5 10880857

[B20] Claverie J Detecting frame shifts by amino acid sequence comparison Journal of molecular biology 1993 234 4 1140 1157 10.1006/jmbi.1993.1666 7903399

[B21] Pellegrini M Yeates T Searching for Frameshift Evolutionary Relationships Between Protein Sequence Families Proteins 1999 37 278 283 10.1002/(SICI)1097-0134(19991101)37:2<278::AID-PROT12>3.0.CO;2-X 10584072

[B22] Arvestad L Aligning coding DNA in the presence of frame-shift errors Proceedings of the 8th Annual CPM Symposium 1997 1264 180 190

[B23] Arvestad L Algorithms for biological sequence alignment PhD thesis 2000 Royal Institute of Technology, Stocholm, Numerical Analysis and Computer Science

[B24] Cornish-Bowden A IUPAC-IUB symbols for nucleotide nomenclature Nucleic Acids Res 1985 13 3021 3030 10.1093/nar/13.9.3021 2582368 PMC341218

[B25] Smith T Waterman M Identification of common molecular subsequences J Mol Bwl 1981 147 195 197 10.1016/0022-2836(81)90087-5 7265238

[B26] Hirschberg D A linear space algorithm for computing maximal common subsequences Communications of the ACM 1975 18 6 341 343 10.1145/360825.360861

[B27] Blake R Hess S Nicholson-Tuell J The influence of nearest neighbors on the rate and pattern of spontaneous point mutations JME 1992 34 3 189 200 10.1007/BF00162968 1588594

[B28] Kosiol C Holmes I Goldman N An Empirical Codon Model for Protein Sequence Evolution Molecular Biology and Evolution 2007 24 7 1464 1479 10.1093/molbev/msm064 17400572

[B29] Lio P Goldman N Models of Molecular Evolution and Phylogeny Genome Research 1998 8 12 1233 1244 9872979 10.1101/gr.8.12.1233

[B30] Pedersen A Jensen J A dependent-rates model and an MCMC-based methodology for the maximum-likelihood analysis of sequences with overlapping reading frames Molecular Biology and Evolution 2001 18 763 776 11319261 10.1093/oxfordjournals.molbev.a003859

[B31] Schneider A Cannarozzi G Gonnet G Empirical codon substitution matrix BMC bioinformatics 2005 6 134 10.1186/1471-2105-6-134 15927081 PMC1173088

[B32] Altschul S Bundschuh R Olsen R Hwa T The estimation of statistical parameters for local alignment score distributions Nucleic Acids Research 2001 29 2 351 361 10.1093/nar/29.2.351 11139604 PMC29669

[B33] Olsen R Bundschuh R Hwa T Rapid assessment of extremal statistics for gapped local alignment Proceedings of the Seventh International Conference on Intelligent Systems for Molecular Biology 1999 AAAI press 211 222 10786304

[B34] Rastas P Salzberg S, Warnow T A General Framework for Local Pairwise Alignment Statistics with Gaps Proceedings of the 9th International Workshop in Algorithms in Bioinformatics (WABI), Philadelphia (USA), of Lecture Notes in Computer Science 2009 5724 Springer Verlag 233 245

[B35] Henikoff S Henikoff J Amino Acid Substitution Matrices from Protein Blocks Proc of the National Academy of Sciences 1992 89 22 10915 10919 10.1073/pnas.89.22.10915 PMC50453 1438297

[B36] Licznar P Bertrand C Canal I Prère MF Fayet O Genetic variability of the frameshift region in IS911 transposable elements from Escherichia coli clinical isolates FEMS Microbiology Letters 2006 218 2 231 237 10.1111/j.1574-6968.2003.tb11522.x 12586397

[B37] Rojas A Garcia-Vallvé S Montero MA Arola L Romeu A Frameshift mutation events in beta-glucosidases Gene 2003 314 191 199 10.1016/S0378-1119(03)00828-X 14527732

[B38] Fry BG Scheib H Weerd L van der Young B McNaughtan J Ryan Ramjan SF Vidal N Poelmann RE Norman JA Evolution of an Arsenal: Structural and Functional Diversification of the Venom System in the Advanced Snakes (Caenophidia) Molecular and Cellular Proteomics 2008 7 215 246 10.1074/mcp.M700094-MCP200 17855442

[B39] Li M Fry BG Kini RM Eggs-Only Diet: Its Implications for the Toxin Profile Changes and Ecology of the Marbled Sea Snake (Aipysurus eydouxii) Journal of Molecular Evolution 2005 60 81 89 10.1007/s00239-004-0138-0 15696370

[B40] Gîrdea M Kucherov G Noé L Salzberg S, Warnow T Back-translation for discovering distant protein homologies Proceedings of the 9th International Workshop in Algorithms in Bioinformatics (WABI), Philadelphia (USA), of Lecture Notes in Computer Science 2009 5724 Springer Verlag 108 120

